# Lager Beer

**Published:** 1860-09

**Authors:** 


### LAGER BEER,

THAT is, "strong" beer is different from other kinds, because it remains longer in a state of fermentation, and just like cider, the longer it stands, the "harder," the stronger, the more alcoholic does it become, the less of it will make a man drunk. Many chemical analyses of lager beer demonstrated that it had "less nutriment and more alcohol than any beer or ale." It has not five per cent of malt, in which lies all the tonic and nutrient qualities. Hence, admitting that beer and ale give any durable nourishment and strength, more lager must be drunk to give a specified amount of tone and vigor, with a great deal more alcohol, than of any other malt liquor. Chemists and certain accommodating doctors have sworn in open court that they believed that a man could not drink lager beer enough to make him drunk, and the impression is sought to be made that it is milder, less hurtful, and more health-giving than other malt drinks, but the truth is, that more alcohol and less nutriment are obtained from lager than from any other beer; hence its habitual use must more certainly impart a taste, a demand, an insatiable craving for strong drink; hence its habitual use is a step towards habitual drunkenness.

